# Epidemiological, Clinical, and Etiological Profile of Iron Deficiency Anemia in Adults in Southern Morocco: A Five-Year Retrospective Study

**DOI:** 10.7759/cureus.116340

**Published:** 2026-09-16

**Authors:** Rida Fatima Ezzahra, Mossaab El Mousadik, Omar Halloumi, Laila Lahlou, Salma Fares

**Affiliations:** 1 Department of Clinical Hematology and Cell Therapy, Mohammed VI University Hospital, Faculty of Medicine and Pharmacy, Ibn Zohr University, Agadir, MAR; 2 Department of Community Medicine, Preventive Medicine, Public Health and Hygiene, Mohammed VI University Hospital, Faculty of Medicine and Pharmacy, Ibn Zohr University, Agadir, MAR

**Keywords:** adults aged 18+, helicobacter pylori, iron-deficiency, iron deficiency anemia (ida), morocco

## Abstract

Background: Iron deficiency anemia is the most common nutritional anemia worldwide, but data on adults in southern Morocco remain limited. This study aimed to describe the epidemiological, clinical, biological, and etiological characteristics of adults with iron deficiency anemia managed at a tertiary referral center in southern Morocco.

Methodology: We conducted a retrospective, descriptive, single-center study of adults aged 18 years or older diagnosed with iron deficiency anemia at the Department of Clinical Hematology and Cell Therapy of the Lalla Salma Regional Oncology Center in Agadir, Morocco, between January 2018 and December 2022. Demographic, clinical, laboratory, and etiological data were collected from medical records.

Results: Among 340 adults evaluated for anemia, 103 (30.3%) had iron deficiency anemia. The median age was 43 years (interquartile range (IQR), 28-54 years), and 78/103 patients (75.7%) were women. Fatigue was the most common presenting symptom (73/103, 70.9%), and mucocutaneous pallor was found on examination in 94/103 patients (91.3%). The mean hemoglobin level was 6.76 ± 1.92 g/dL, and anemia was severe in 77/103 patients (74.8%). The median serum ferritin level was 3.8 µg/L (IQR, 2.0-7.0). An underlying etiology was identified in 74/103 patients (71.8%). Blood loss was the leading mechanism (40/103, 38.8%), predominantly gynecological (30/78, 38.5%), followed by impaired iron absorption (27/103, 26.2%), mainly related to *Helicobacter pylori* infection (23/103, 22.3%).

Conclusion: Among adults with iron deficiency anemia managed in this tertiary hematology referral setting, women predominated, and severe anemia was frequent at presentation. Gynecological and gastrointestinal causes were the most frequently documented etiologies. Appropriate etiological evaluation is essential to improve management.

## Introduction

Iron deficiency is the most common nutritional cause of anemia, and iron deficiency anemia is the most prevalent form of anemia worldwide. It particularly affects children and women of reproductive age [1,2]. In 2021, anemia affected approximately 1.92 billion people worldwide, corresponding to an overall prevalence of 24.3% [3]. Biochemical confirmation of iron deficiency and identification of its underlying cause, particularly blood loss, malabsorption, or gastrointestinal disease, are essential steps in the management of affected patients [4,5]. In Morocco, the National Nutrition Survey (2019-2020) reported, among women of reproductive age, an anemia prevalence of 34.4% and, separately, an iron deficiency prevalence of 30.3% at the population level [6]. However, hospital-based data describing adults with iron deficiency anemia requiring specialist hematology evaluation, particularly in southern Morocco, remain limited. This study aimed to describe the epidemiological, clinical, biological, and etiological characteristics of adults with iron deficiency anemia managed at a tertiary hematology referral center in southern Morocco and to estimate the proportion of iron deficiency anemia among adults investigated for anemia within this specialized setting during the study period.

## Materials and methods

Study design and population

This was a descriptive, retrospective, single-center study conducted from January 1, 2018, to December 31, 2022, in the Department of Clinical Hematology and Cell Therapy, which was temporarily based at the Lalla Salma Regional Oncology Center in Agadir, a tertiary referral center in southern Morocco, pending the opening and operationalization of Mohammed VI University Hospital in Agadir. All patients aged 18 years or older with iron deficiency anemia, including pregnant women, were eligible for inclusion. Patients with iron deficiency without anemia, vitamin B9 or vitamin B12 deficiency, or mixed nutritional anemia were excluded. Demographic, clinical, laboratory, and etiological data were retrospectively collected from medical records using a standardized data collection form.

Definitions

According to the World Health Organization (WHO) criteria applicable during the study period, anemia was defined as a hemoglobin level below 13 g/dL in men, below 12 g/dL in nonpregnant women, and below 11 g/dL in pregnant women [7]. Anemia severity was graded according to the same WHO criteria as severe (hemoglobin <8 g/dL), moderate (8.0-10.9 g/dL), or mild (11.0-11.9 g/dL in nonpregnant women and 11.0-12.9 g/dL in men); in pregnant women, thresholds of <7.0 g/dL, 7.0-9.9 g/dL, and 10.0-10.9 g/dL were applied, respectively [7].

Serum ferritin was the primary marker used to assess iron stores. In adults without inflammation, iron deficiency was defined as a serum ferritin concentration <15 µg/L, according to WHO recommendations [8]. Because inflammatory markers and additional iron studies were not systematically available in this retrospective cohort, the potential influence of inflammation on ferritin interpretation could not be assessed uniformly in all patients. No patient included in the final sample had a ferritin level above 29 µg/L. Among pregnant women, no trimester-specific ferritin threshold was applied because the trimester of pregnancy was not consistently documented in the medical records. Geophagic pica was defined as the recurrent ingestion of soil or earth-containing substances [9].

An etiology was considered documented when a cause of iron deficiency was identified by the diagnostic work-up performed during the index episode. Because of the retrospective design, etiological investigations were guided by the clinical context and were not performed according to a uniform standardized protocol. Etiologies were classified into three mechanisms: blood loss, impaired iron absorption, and increased iron requirements or inadequate dietary intake.

Statistical analysis

Statistical analysis was performed using jamovi version 2.6.26 (https://www.jamovi.org). Categorical variables were expressed as frequencies and percentages. The normality of continuous variables was assessed using Q-Q plots and the Shapiro-Wilk test. Normally distributed continuous variables were expressed as mean ± standard deviation, whereas non-normally distributed variables were reported as median with interquartile range (IQR, Q1-Q3). Unless otherwise specified, percentages were calculated using the whole study population (n = 103) as the denominator; sex-specific percentages were calculated using the corresponding sex-specific denominators (n = 78 for women and n = 25 for men).

## Results

Study population

During the five-year study period, 340 adults were evaluated for anemia, of whom 103 (30.3%) met the criteria for iron deficiency anemia and were included in the analysis (Figure 1).

**Figure 1:**
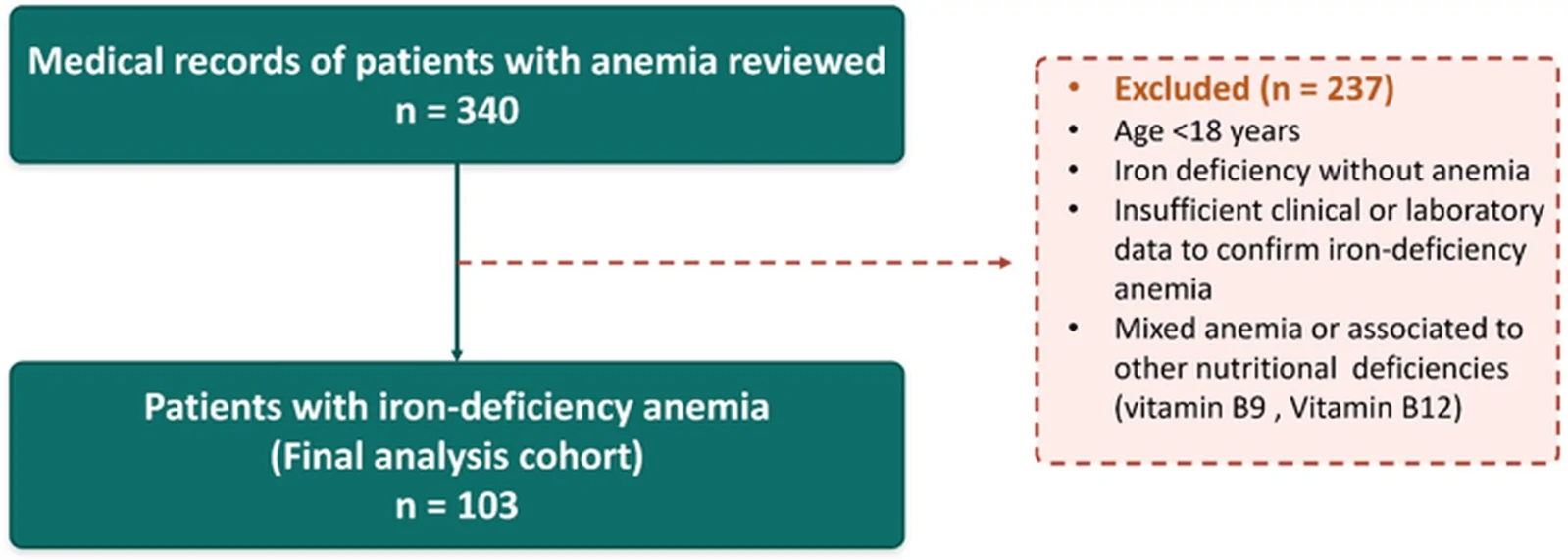
Flow diagram

The median age was 43 years (IQR, 28-54 years; range, 18-90 years). Most patients were aged 18-50 years (75/103, 72.8%). Women predominated, accounting for 78 patients (75.7%), with a male-to-female ratio of 0.32. A total of 63 patients (61.2%) lived in urban areas, 68 (66.0%) had a low socioeconomic status, and 31 (30.1%) had no formal education (Table 1).

**Table 1:** Sociodemographic characteristics of the study population (n = 103) *Values are presented as median (interquartile range). **Values are presented as counts and percentages (%).

Characteristic	Value (n = 103)
Age, years*	43 (28-54)
Age range, years	18-90
Age group, years**:	
18-30	29 (28.2)
31-40	14 (13.6)
41-50	32 (31.1)
51-60	11 (10.7)
61-70	6 (5.8)
>70	11 (10.7)
Female sex**	78 (75.7)
Male sex**	25 (24.3)
Male-to-female ratio	0.32
Urban residence**	63 (61.2)
Low socioeconomic status**	68 (66.0)
No formal education**	31 (30.1)

Risk factors

Among the 78 women, parity of at least one was reported in 34 (43.6%), menorrhagia or menometrorrhagia in 28 (35.9%), a history of abortion in 7 (8.9%), and current pregnancy in 4 (5.1%). In the overall study population, geophagic pica and excessive tea consumption were each reported in 9 patients (8.7%), while 6 patients (5.8%) had a history of gastrointestinal disease. A vegetarian diet was reported in 1 patient (1.0%) (Table 2).

**Table 2:** Risk factors for iron deficiency anemia in the study population **Values are presented as counts and percentages (%). Gynecological and obstetric factors were calculated among women only (n = 78); other factors were calculated in the entire study population (n = 103).

Characteristic	Value
Gynecological and obstetric factors**	n = 78
Parity >=1	34 (43.6)
Menorrhagia or menometrorrhagia	28 (35.9)
History of abortion	7 (8.9)
Current pregnancy	4 (5.1)
Other risk factors**	n = 103
Geophagic pica	9 (8.7)
Excessive tea consumption	9 (8.7)
History of gastrointestinal disease	6 (5.8)
Vegetarian diet	1 (1.0)

Clinical findings

Fatigue was the most common presenting complaint, reported by 73 patients (70.9%), followed by self-reported mucocutaneous pallor in 51 (49.5%), exertional dyspnea in 16 (15.5%), and palpitations in 8 (7.8%). On physical examination, mucocutaneous pallor was observed in 94 patients (91.3%), while skin and appendage abnormalities were present in 27 (26.2%). Sinus tachycardia was documented in 8 patients (7.8%).

Overt bleeding at the time of presentation was identified in 12 patients (11.7%), including menorrhagia (5 cases), rectal bleeding (3 cases), gingival bleeding (2 cases), epistaxis (1 case), and melena (1 case).

Laboratory findings

The mean hemoglobin level was 6.76 ± 1.92 g/dL (range, 2.7-11.4 g/dL). According to WHO severity criteria applied by sex and pregnancy status, anemia was severe in 77 patients (74.8%), moderate in 23 (22.3%), and mild in 3 (2.9%). Hemoglobin was below 6 g/dL in 36 patients (35.0%).

The mean corpuscular volume was 66.70 ± 8.00 fL (range, 48-88 fL), and the mean corpuscular hemoglobin concentration was 27.04 ± 2.83 g/dL (range, 21.0-34.4 g/dL). Thrombocytosis, defined as a platelet count above 450,000/mm³, was observed in 22 patients (21.4%). The median serum ferritin level was 3.8 µg/L (IQR, 2.0-7.0 µg/L; range, 1-29 µg/L). The median platelet count was 288,000/mm³ (IQR, 183,500-419,500/mm³), and the median white blood cell count was 6,240/mm³ (IQR, 5,000-8,750/mm³) (Table 3). Eosinophil counts were not systematically available in the retrospective records and were therefore not included in the analysis.

**Table 3:** Laboratory Characteristics at Diagnosis (n = 103) *Values are presented as median (interquartile range). **Values are presented as counts and percentages (%). †Values are presented as mean ± standard deviation.

Characteristic	Value (n = 103)
Hemoglobin, g/dL†	6.76 ± 1.92
Hemoglobin <6 g/dL**	36 (35.0)
Severe anemia**	77 (74.8)
Moderate anemia**	23 (22.3)
Mild anemia**	3 (2.9)
Mean corpuscular volume, fL†	66.70 ± 8.00
Mean corpuscular hemoglobin concentration, g/dL†	27.04 ± 2.83
Serum ferritin, µg/L*	3.8 (2.0-7.0)
Thrombocytosis (>450,000/mm³)**	22 (21.4)
Platelet count, /mm³*	288,000 (183,500-419,500)
White blood cell count, /mm³*	6,240 (5,000-8,750)

Etiological findings

An underlying etiology was identified in 74/103 patients (71.8%), including 64/78 women (82.1%) and 10/25 men (40.0%). Blood loss was the most frequent mechanism, identified in 40/103 patients (38.8%), corresponding to 54.1% of the patients with a documented etiology. Gynecological bleeding occurred exclusively among women and was documented in 30/78 women (38.5%).

Gastrointestinal blood loss was documented in 9/103 patients (8.7%), and was more frequent in men (6/25, 24.0%) than in women (3/78, 3.8%). The identified causes were inflammatory bowel disease (3/103, 2.9%), colorectal cancer (2/103, 1.9%), bleeding internal hemorrhoids (2/103, 1.9%), esophageal varices (1/103, 1.0%), and colonic angiodysplasia (1/103, 1.0%). Ear, nose, and throat blood loss, in the form of recurrent epistaxis, accounted for 1/103 cases (1.0%).

Impaired iron absorption was identified in 27/103 patients (26.2%), corresponding to 36.5% of the patients with a documented etiology, and included 24/78 women (30.8%) and 3/25 men (12.0%). Helicobacter pylori infection was the predominant cause (23/103, 22.3%), while celiac disease accounted for 4/103 cases (3.9%). Increased iron requirements or inadequate dietary iron intake accounted for 7/103 patients (6.8%), all of them women, and comprised current pregnancy in 3/103 (2.9%) and inadequate dietary iron intake in 4/103 (3.9%) (Table 4).

**Table 4:** Etiological profile of iron deficiency anemia according to sex Values are presented as counts and percentages (%). Percentages in the "total" column are calculated using the entire study population (n = 103), whereas percentages in the "women" and "men" columns are calculated using the respective sex-specific denominators (n = 78 and n = 25).

Mechanism and cause	Total (n = 103)	Women (n = 78)	Men (n = 25)
Blood loss	40 (38.8)	33 (42.3)	7 (28.0)
Gynecological blood loss	30 (29.1)	30 (38.5)	0
Functional menometrorrhagia	22 (21.4)	22 (28.2)	0
Uterine fibroids/polymyomatous uterus	6 (5.8)	6 (7.7)	0
Endometrial polyp	1 (1.0)	1 (1.3)	0
Cervical cancer	1 (1.0)	1 (1.3)	0
Gastrointestinal blood loss	9 (8.7)	3 (3.8)	6 (24.0)
Inflammatory bowel disease	3 (2.9)	1 (1.3)	2 (8.0)
Colorectal cancer	2 (1.9)	0	2 (8.0)
Bleeding internal hemorrhoids	2 (1.9)	1 (1.3)	1 (4.0)
Esophageal varices	1 (1.0)	0	1 (4.0)
Colonic angiodysplasia	1 (1.0)	1 (1.3)	0
Ear, nose, and throat blood loss (recurrent epistaxis)	1 (1.0)	0	1 (4.0)
Impaired iron absorption	27 (26.2)	24 (30.8)	3 (12.0)
*Helicobacter pylori *infection	23 (22.3)	20 (25.6)	3 (12.0)
Celiac disease	4 (3.9)	4 (5.1)	0
Increased iron requirements or inadequate intake	7 (6.8)	7 (9.0)	0
Current pregnancy	3 (2.9)	3 (3.8)	0
Inadequate dietary iron intake	4 (3.9)	4 (5.1)	0
Documented etiology	74 (71.8)	64 (82.1)	10 (40.0)
No etiology documented	29 (28.2)	14 (17.9)	15 (60.0)

## Discussion

Our study, one of the first hospital-based series focusing on iron deficiency anemia in adults managed in a tertiary hematology setting in southern Morocco, highlights a female predominance, a relatively young population, and a frequent presentation with severe anemia among referred patients. The low mean hemoglobin concentration (6.76 g/dL) and the marked depletion of iron stores (median ferritin, 3.8 µg/L) reflect the severity of iron deficiency among patients evaluated at our center. Importantly, the high proportion of severe anemia should be interpreted in the context of tertiary-care referral and may partly reflect referral selection rather than the timing of diagnosis in the wider population. The female predominance (75.7%) and the concentration of cases in the 18-50-year age range (72.8%) are consistent with findings reported in the literature, as women of reproductive age are particularly vulnerable because of menstrual blood loss and increased iron requirements [2,10]. The clinical manifestations observed, predominantly fatigue (70.9%) and mucocutaneous pallor (91.3%), and the thrombocytosis observed in 21.4% of patients are also consistent with published descriptions of iron deficiency anemia [2,11]. Although thrombocytosis is well described in iron deficiency anemia, its underlying mechanism could not be specifically assessed in this retrospective cohort.

Iron deficiency anemia accounted for 30.3% of the adults investigated for anemia within our tertiary hematology service during the study period. This proportion describes the distribution of iron deficiency anemia within this selected hospital-based population and should not be interpreted as a population-level prevalence of iron deficiency anemia in southern Morocco or confused with the population-level prevalence of iron deficiency reported by the Moroccan National Nutrition Survey among women of reproductive age, nor with global estimates of anemia in women aged 15-49 years [6,12]. In an internal medicine department in Meknes, Morocco, Zinebi et al. reported iron deficiency anemia in 60% of 150 patients investigated for anemia, with a mean hemoglobin level of 8 g/dL, a mean age of 48.8 years, and a female predominance [13]. The lower proportion observed in our series is plausibly explained by our recruitment in a hematology and oncology referral center, where anemia related to malignancy and chronic disease is over-represented. Similarly, the lower mean hemoglobin level in our patients (6.76 vs 8 g/dL) indicates greater severity among patients evaluated at our tertiary center but cannot be interpreted as evidence of delayed diagnosis in the wider population.

Blood loss was the leading identified etiological mechanism, followed by impaired iron absorption. Gynecological blood loss predominated among hemorrhagic causes, with functional menometrorrhagia being the most frequent single cause. Gastrointestinal blood loss included inflammatory bowel disease, colorectal cancer, bleeding internal hemorrhoids, esophageal varices, and colonic angiodysplasia, and was markedly more frequent in men (24.0%) than in women (3.8%). However, this sex-related difference should be interpreted cautiously because an etiology remained undocumented in 60.0% of men compared with 17.9% of women. Nevertheless, these findings support an etiological evaluation tailored to sex, age, and clinical context, and in particular the endoscopic evaluation recommended in men and postmenopausal women by the American Gastroenterological Association (AGA) and British Society of Gastroenterology (BSG) guidelines [4,5].

Helicobacter pylori infection was the most frequently identified condition associated with impaired iron absorption in our series, followed by celiac disease. A systematic review and meta-analysis has shown that infected individuals have a higher risk of iron deficiency anemia than uninfected ones, and that eradication therapy combined with oral iron improves hemoglobin and ferritin levels more than iron therapy alone [14]. In a country with a high background prevalence of this infection, and in line with the recommended approach to unexplained or refractory iron deficiency anemia, investigation for H. pylori infection and celiac disease appears appropriate when no bleeding source is identified [5,15].

An underlying etiology could not be documented in 29 patients (28.2%), and this proportion was markedly higher in men (60.0%) than in women (17.9%). This finding reflects incomplete etiological assessment within our study population and should not be interpreted as evidence of difficulties in the diagnostic pathway across southern Morocco. Because referral history, previous investigations, and the reasons for incomplete etiological assessment were not systematically recorded, the pathway preceding tertiary referral could not be evaluated. Iron supplementation should not, however, replace investigation of the underlying cause, particularly in adults in whom an underlying gastrointestinal disorder may present with iron deficiency anemia [4,5].

In our population, excessive tea consumption was reported in 9 patients (8.7%), while inadequate dietary iron intake was identified in four patients (3.9%). Polyphenol-rich beverages such as tea, which is widely consumed with meals in Morocco, inhibit non-heme iron absorption in a dose-dependent manner and may reduce it by 60% to 90% when consumed with a meal [16]. These observations highlight the importance of appropriate etiological evaluation, dietary counseling, and multidisciplinary management in patients referred with iron deficiency anemia.

Limitations

This study provides valuable hospital-based data on adults with iron deficiency anemia managed in a tertiary referral setting in southern Morocco. Its main limitations include its retrospective and single-center design and recruitment from a tertiary hematology service, which introduces referral and selection bias and limits the generalizability of our findings. The high proportion of severe anemia may therefore partly reflect the characteristics of patients reaching tertiary care rather than the severity of iron deficiency anemia in the wider regional population. Referral source, previous management in primary or secondary care, the timing of initial diagnosis, and the specific reasons for tertiary referral were not systematically documented and could not be assessed.

The etiological assessment was incomplete in 29 patients, particularly among men, in whom no etiology was documented in 60.0% of cases; this limits the interpretation of sex-specific etiological differences. The trimester of pregnancy was not consistently documented, and no therapeutic follow-up or outcome data were available. Finally, etiological investigations were not performed according to a uniform standardized protocol, and the absence of systematic endoscopic and gynecological investigation may have led to underestimation of some causes. Inflammatory markers and additional iron studies were also not systematically available in this retrospective cohort. Prospective multicenter studies, including different levels of care and standardized etiological evaluation, are warranted to better characterize iron deficiency anemia in adults in Morocco.

## Conclusions

Our study shows that, among adults with iron deficiency anemia managed in a tertiary hematology referral setting in southern Morocco, women predominated, and severe anemia was frequent at presentation. Gynecological blood loss, gastrointestinal blood loss, and conditions associated with impaired iron absorption were the most frequently documented etiologies, while no underlying cause was identified in more than one-quarter of patients. These findings characterize the subset of patients reaching specialist hematology care and should not be extrapolated to the general population or to primary-care pathways in the region. They highlight the importance of appropriate etiological evaluation and multidisciplinary management in referred patients. Prospective multicenter studies including different levels of care would help to better characterize iron deficiency anemia in adults in Morocco and optimize its management.
